# Armed conflict and mental health in Colombia

**DOI:** 10.1192/bji.2018.4

**Published:** 2019-05

**Authors:** William Tamayo-Agudelo, Vaughan Bell

**Affiliations:** 1 Profesor de Psicologia, Universidad Cooperativa de Colombia, Medellín, Colombia; 2 Division of Psychiatry, University College London, UK; 3 Senior Clinical Lecturer, Division of Psychiatry, University College London, UK, email vaughan.bell@ucl.ac.uk; 4 South London and Maudsley National Health Service Foundation Trust, UK

## Abstract

Although significant progress has been made in the peace process, Colombia still experiences high levels of ongoing violence and a legacy of more than five decades of armed conflict. Epidemiological studies show markedly raised levels of mental health problems in people affected by the conflict, with internally displaced people being a large and important group with unmet needs. Provision of mental health services is uneven and subject to significant underinvestment. Priority mental health treatment for victims of the conflict is now established in law, although the effectiveness of these programmes has yet to be established.

The Colombian armed conflict has continued for over 50 years and has left approximately 220 000 people dead, 6 million displaced and more than 27 000 kidnapped; leading to huge social and economic costs to the country, and massive personal costs to those affected (Grupo de Memoria Histórica, 2016). The recent demobilisation of the Revolutionary Armed Forces of Colombia (FARC-EP) following the 2016 peace accord has reduced the overall intensity of the conflict. However, some areas maintain high levels of violence due to hostilities between numerous groups, including state actors, remaining and dissident revolutionary guerrilla groups, right-wing paramilitaries and criminal drug trafficking organisations. For the civilian population, the conflict has been characterised by frequent and extensive forced displacements, violent control of communities, forced labour, targeted killings and massacres, disappearances, sexual violence, extortion, corruption and the systemic embedding of violence within community life.

Political debate surrounding the peace process has led to marked social and political polarisation. Key points of disagreement include justice and compensation for those affected by the conflict, integration of increasing numbers of demobilised guerrillas and government response to ongoing violence. Mental health has become part of this debate because of the direct effects of the conflict on the population as well as the challenges faced by mental health services in Colombia.

## Exposure to the armed conflict as a predictor of mental health problems

Although Colombia's armed conflict is often described as ‘low intensity’, independent data suggest remarkably high levels of exposure to conflict-related violence in the civilian population. Gómez-Restrepo *et al* (2016a) examined this using two data sources: the 2015 National Mental Health Survey (NMHS), which reports formally sampled epidemiological data stratified by region (Atlántica, Oriental, Central, Pacífica and Bogotá) covering both urban and rural populations and including participants aged 7 years and older, and CERAC, which is an independent register of conflict-related violence. Experience of permanent conflict (defined as the presence of armed groups) was reported by 47.2% of individuals and in 21.8% of municipalities, and intermittent conflict was reported by 44.1% of individuals and in 65.5% of municipalities. Anxiety, mood disorders and suicide were elevated in areas with a higher constancy and intensity of conflict. However, possible post-traumatic stress disorder was most frequent in areas with lower intensity and intermittent conflict, which potentially reflects the persistence of trauma-related experiences after periods of more intense violence.

## Impact and nature of forced displacement

More people have been displaced by violence in Colombia than in any other country in the world (Shultz *et al*, 2014), and the mental health needs of internally displaced people are central to understanding the effects of the armed conflict. A 2014 systematic review of mental health in displaced people found high levels of symptoms (range of 9.9–63%) and diagnosable disorders (1.5–32.9%), but large variability due to the relatively poor quality of studies (Campo-Arias *et al*, 2014). Later, a study by Tamayo Martínez *et al* (2016) (using the 2015 NMHS data) reported a 15.9% lifetime prevalence of diagnosable psychiatric disorders in displaced adults. Although this study did not report a direct statistical comparison with non-displaced individuals, the baseline rate for adults in Colombia using the same data set is 10.1% (Gómez-Restrepo *et al*, 2016b), suggesting a higher prevalence of mental health problems in displaced adults.

Importantly, the impact of forced displacement on mental health is likely to arise from multiple sources over a protracted period. To capture this, Shultz *et al* (2014) applied trauma signature analysis to profile forced displacement in Colombia. This is a systematic method for identifying the features of a natural or human-generated disaster that characterises stages, hazards, stressors and impact to better guide effective mental health and psychosocial support. They found that forced displacement in Colombia typically involves already-vulnerable groups fleeing violence in rural areas, risking violence and exploitation during migration, and then settling in areas on the outskirts of large cities which are often under the control of armed groups. It is also notable that essential services such as sanitation, electricity, health, transport and education are often slow to extend to these areas, and social problems such as criminal exploitation, high levels of drug use and gender-based violence are more common. Furthermore, displaced people often face significant social stigma and women, children and already-marginalised groups (for example, African–Colombian citizens) are over-represented.

## Impact on children and adolescents

The impact of the armed conflict on children and adolescents is still poorly understood. Published analyses of the 2015 NMHS data on children (Gómez-Restrepo *et al*, 2016c) and adolescents (Gómez-Restrepo *et al*, 2016d) found that displacement by violence was not associated with a significantly increased chance of meeting the criteria for psychiatric diagnosis, although past trauma was a strong and significant predictor (odds ratio: 4.2; 95% CI: 2.3–7.8). Previous studies on smaller samples typically report that exposure to conflict or community or domestic violence is a clear predictor of mental health problems and behavioural difficulties. For example, Flink *et al* (2013) investigated mental health problems in preschool children in Bogotá and found markedly higher rates of problems (odds ratio: 3.3; 95% CI: 1.5–6.9) in children from families displaced by violence.

## Current challenges

Colombia faces a unique combination of challenges with respect to mental health. Adequate services need to be available to (a) the population as a whole, as they have traditionally had poor access to mental health services and have lived with internationally high levels of systemic violence for many decades; (b) people displaced by the conflict, as they make up almost 15% of the Colombian population and have additional needs but often live in communities with further risk factors for poor mental health and lack of access to support; and (c) individuals with very high exposure to the conflict, as they may have more severe and complex problems that require specialist treatment. This latter group includes civilian victims of violence, torture and other human rights abuses but also includes combatants and ex-combatants from armed groups who need to be reintegrated into society. Combatants may also have been both victims and perpetrators of human rights abuses, leading to complex care needs that involve balancing personal well-being, public protection and political acceptability.

However, uneven availability of services and relatively low levels of investment in mental health and the mental health workforce are still major obstacles (Chaskel *et al*, 2015). Mental health services are most widely available in urban centres and can be either be absent or sparse in the rural areas most affected by the conflict. Although Colombia provides almost universal healthcare coverage, the current two-tier system provides a markedly poorer level of care for people on the government-subsidised system. In addition, corruption, stalled reforms, health system debts and closure of mental health hospitals and clinics are significant barriers to progress.

One important step has been the development of a national programme that prioritises healthcare for people affected by the conflict, and mental health provision plays a central role in this programme. Law 1448 (2011), passed in 2011, established a programme for social and psychological support for victims of the armed conflict (*Programa de atención psicosocial y salud integral a víctimas*; PAPSIVI) that is based on the principles of human rights, public health and community psychology. The programme is intended to significantly increase access to mental health services for those affected by the armed conflict, mainly through community physicians, psychologists and social workers.

Although PAPSIVI is a promising and well-designed programme, it is in its early stages and concerns have been raised about slow implementation and future capacity to provide services to millions of people (Sánchez Jaramillo, 2016). Initial concerns about a lack of evidence-based recommendations for interventions and unclear standards for clinicians have been partly addressed by the publication of the 2017 PAPSIVI protocol manual (*Protocolo de Atención Integral en Salud con Enfoque Psicosocial a Víctimas del Conflicto Armado*) (MinSalud-ITES, 2017). However, a lack of standardised methods for measuring outcome remains a limitation in evaluating the effectiveness of the programme. It is also notable that this protocol has only recently become available and it is not clear to what extent these standards are being successfully implemented in existing teams.

## Conclusions

Colombia has significant challenges in addressing the scale of conflict-related mental health needs. These challenges include the need for a sufficient evidence base to characterise needs and guide interventions, and adequate mental health services to provide support. Although evidence is lacking across the board, the mental health of children and adolescents is particularly under-researched and should be a priority. Notably, the NMHS is an excellent but underused resource and studies using this data set could answer many key questions relating to mental health policy. There is also a severe lack of research evaluating the effectiveness of interventions and this research should be made a priority.

In terms of existing services, PAPSIVI is a promising component despite concerns about its implementation and evaluation. However, it is only available to those who are registered or able to register as victims of the armed conflict. Better integration of mental healthcare into primary care may be an additional step that has the potential to address the wider systemic effects of armed conflict on mental health (Rodríguez de Bernal & Rubiano Soto, 2016).
